# Supplementary material for: Investigating the accuracy of blood oxygen saturation measurements in common consumer smartwatches
Source: PLOS Digit Health. 2023 Jul 12;2(7):e0000296. doi: 10.1371/journal.pdig.0000296 (PMC10337940; doi:10.1371/journal.pdig.0000296)
Supplement: S1 Text — (DOCX) [file pdig.0000296.s001.docx]

| Device | Disclaimer | Factors that affect the accuracy |
| --- | --- | --- |
| Apple Watch Series 7 | Blood Oxygen app measurements are not intended for medical use, including self-diagnosis or consultation with a doctor, and are only designed for general fitness and wellness purposes.[1] | Skin perfusion, permanent or temporary changes to your skin, motion, certain postures, too-high heart rate.[1] |
| Garmin Venu 2 | The Pulse Ox data is not intended to be used for medical purposes, nor is it intended to diagnose, treat, cure or prevent any disease or condition. [2] | Physical characteristics, fit of the device, and presence of ambient light.[2] |
| Garmin Fenix 6 Pro | Same as for Garmin Venu 2 | The same as Garmin Venu 2 |
| Withings ScanWatch | Even under ideal conditions, a SpO_2_ measurement may not be conclusive. An inconclusive result may be caused by inaccurate data or an element affecting the process. [3] | Bright sunlight, presence of strong electromagnetic fields, failure to apply the device correctly (loose wristband application, not on top of the wrist), tattoos on the wrist in the region of the optical sensor, excessive motion of the arm, wrist, or fingers, low perfusion caused by ambient temperature below the recommended operation range, or by certain conditions such as Raynaud’s syndrome, significant levels of dysfunctional hemoglobin (carboxyhemoglobin, methemoglobin), venous pulsations Intravascular dyes such as cardio green or methyl blue, blood-flow restrictions due to arterial catheters, blood pressure cuffs, or infusion lines, and hypotension, serious vasoconstriction, serious anemia, or hypothermia Cardiac arrest or shock. [4] |

1.How to use the Blood Oxygen app on Apple Watch Series 6 or Series 7 [Internet]. Apple Support. [cited 2022 Feb 1]. Available from: https://support.apple.com/en-us/HT211027

2. Accuracy | Garmin [Internet]. [cited 2022 Feb 1]. Available from: https://www.garmin.com/en-US/legal/atdisclaimer/

3. ScanWatch - Performing a SpO2 measurement [Internet]. Withings | Support. [cited 2022 Feb 1]. Available from: https://support.withings.com/hc/en-us/articles/360010097498-ScanWatch-Performing-a-SpO2-measurement

4. ScanWatch - Important information about SpO2 measurements [Internet]. Withings | Support. [cited 2022 Feb 1]. Available from: https://support.withings.com/hc/en-us/articles/360010332577-ScanWatch-Important-information-about-SpO2-measurements
