# Supplementary figures and images for: Investigating the accuracy of blood oxygen saturation measurements in common consumer smartwatches
Source: PLOS Digit Health. 2023 Jul 12;2(7):e0000296. doi: 10.1371/journal.pdig.0000296 (PMC10337940; doi:10.1371/journal.pdig.0000296)

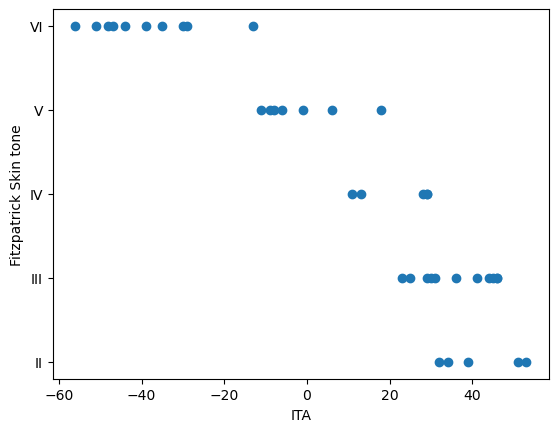

Supplement: S1 Fig — (PNG) [file pdig.0000296.s006.png]
