# Supplementary material for: Investigating the accuracy of blood oxygen saturation measurements in common consumer smartwatches
Source: PLOS Digit Health. 2023 Jul 12;2(7):e0000296. doi: 10.1371/journal.pdig.0000296 (PMC10337940; doi:10.1371/journal.pdig.0000296)
Supplement: Attachment — Submitted filename: SpO2 paper rebuttal from PLOS Digital Health.pdf [file pdig.0000296.s007.pdf]

Response to Reviewers

Thank you for the efforts to review our work. We appreciate the thoughtful and pertinent feedback, and have implemented changes that have substantially improved the manuscript as outlined below.

—

Reviewer #1: This study compares the accuracy and reliability of SpO2 measurements from consumer smart watches to a commercially available pulse oximeter. This is an important topic as the popularity and availability of these devices is growing. I offer the following major and minor comments for consideration:

Major comments

- A wide range of SpO2 measurements were reported (82.0-100.0%) and no explanation is offered for the range of results outside normal values. Co-morbidities such as participant history of respiratory disease should be reported if known. Furthermore, if any of the participants were receiving oxygen therapy this should be reported. It is also unclear in the methodology if the patients were inpatients or outpatients. This would allow for interpretation of the results in the various clinical scenarios. The authors should also offer any possible explanations for the variation in oxygen saturations reported.

We thank the reviewer for this suggestion and agree that further explanation for the range of SpO_2 measurements would add merit to this paper. We have updated the Patient Cohort section within the Results section to include the subjects' medical history, including pulmonary or cardiovascular disease, heart or lung transplant history, whether subjects were inpatient or outpatient, as well as any oxygen use.

- This study compares the accuracy and reliability of pulse oximetry from consumer smart watches to a commercially available pulse oximeter. Both technologies are based on the absorption of light at different wavelengths. However, it has been shows that there are racial biases in the pulse oximetry measurements, when compared to the gold standard of arterial blood gas measurements. A significant limitation of this study is that the consumer watch pulse oximeters are directly compared to another device that has known racial biases, therefore this bias is not accounted for in this study when measuring the reliability and accuracy of the smart watches. Furthermore, the accuracy of pulse oximeters is also influenced by several other factors such as poor perfusion, anemia and hemoglobinopathies. These limitations should be acknowledged and discussed in the manuscript.

We agree that an inherent limitation of this study is the reference device. We chose to use this device because it is the current standard of care used in clinics and therefore is currently the most common method that clinicians use for decision-making. We agree with the reviewer that it remains important to truly understand the potential limitations of the current clinical oximeters

and have discussed this potential issue of racial bias and its possible effects on our study's findings on lines 91-96, 334-340. Further, a future clinical study that uses the more invasive arterial blood gas measurements as a reference standard would help to address this issue, but this is out of scope of the current study.

Additionally, we appreciate the reviewer highlighting factors that could influence pulse oximeter accuracy that should be addressed in this paper. For this study, we excluded patients with peripheral vascular disease, Raynaud's syndrome, cryoglobulinemia, or any collagen vascular disease affecting the fingers, a history of blood clots in the last 6 months, an essential tremor, or gel nail polish or any other non-natural, non-removable discoloration of the forefinger. This information is reported in the first paragraph of the Materials and Methods section. Regarding the factor of poor perfusion, we attempted to exclude these patients by screening for peripheral vascular disease, which was one of the exclusion criteria. Regarding anemia, this was not one of our exclusion criteria, but there is ongoing research into the effect it has on pulse oximetry. Several papers (Ortiz et al., 1999; Fitzgerald et al., 2001; Perkins et al., 2003) and one case study (Hinkelbein et al., 2004) on patients with anemia and sickle cell anemia all concluded that, although there is some variability in the relationship between SaO_2 and SpO_2, this was not a clinically significant difference, and that pulse oximetry should remain an important diagnostic tool in these patients. Regarding hemoglobinopathies, we did not screen for these in our study. We have added these factors into the discussion of limitations in the manuscript, under the Discussion section.

[1] Ortiz FO, Aldrich TK, Nagel RL, Benjamin LJ. Accuracy of Pulse Oximetry in Sickle Cell Disease. Am J Respir Crit Care Med. 1999 Feb;159(2):447–51.
[2] Fitzgerald RK, Johnson A. Pulse oximetry in sickle cell anemia. Critical Care Medicine. 2001 Sep;29(9):1803.
[3] Perkins GD, McAuley DF, Giles S, Routledge H, Gao F. Do changes in pulse oximeter oxygen saturation predict equivalent changes in arterial oxygen saturation? Critical Care. 2003 Jun 11;7(4):R67.
[4] Hinkelbein J, Osika A, Fiedler F. Accuracy of pulse oximetry in a case of severe anemia from gastrointestinal hemorrhage: A-167. European Journal of Anaesthesiology | EJA. 2004 Jun;21:42.

- The Fitzpatrick skin phototypes is a scale that was designed to assess a person's risk of developing sunburn, although it is often used as a proxy for race/ethnicity. The scale is disproportionally representative of White skin types. Additionally, the authors report the breakdown of included races as only Black or White race. The limitations and biases of the FST scale as well as the lack of inclusion of participants from other races should be acknowledged and discussed by the authors.

The Fitzpatrick scale, while an industry standard, is indeed not the best suited tool for the task at hand. We have clarified its limitations on lines 342-348 of the Discussion section. To address the limitations of the Fitzpatrick scale, in this study we also added objectively-measured skin tone using an optical measurement tool, the *Delfin SkinColorCatch,* which reports the Individual

Typology Angle (ITA). In the supplement of our paper, we show the high concordance between the Fitzpatrick and ITA measurements. Further, we evaluated whether ITA impacted the outcomes of interest for this study, including the MAE, MDE, Missingness and RMSE between SpO_2 readings from smartwatches and reference standard.

Minor comments

- The authors should include references in the second paragraph of the introduction (lines 63-74) to support their statements about the use of pulse oximetry in COVID-19 disease and the use of home oxygen saturation monitoring.

We have addressed this issue in the updated manuscript (now on lines 67 - 75).

- Line 85-87 the authors discuss that they have recently assessed the accuracy of heart rate measurements from smartwatches. A reference should be provided for this.

We have addressed this issue in the updated manuscript (now on lines 85 - 87).

- The authors report missing data for the ITA scale for 11/49 patients. It would be helpful to report the Fitzpatrick data for the missing ITA measurements to allow readers a more comprehensive assessment of the missing data and potential biases. For example, were the missing ITA measurements predominantly one end of the spectrum of the Fitzpatrick scale?

We would like to thank the reviewer for pointing it out. We add the Fitzpatrick data for the missing ITA measurements in the Supplementary Material Table (Table S5). The missingness of the ITA measurements was not a technical issue but rather the result of the late arrival of our equipment, which therefore had no systematic impact on the study results other than resulting in missing data in the first 11 participants. As such, the missingness of the ITA data is not related to the subjects' skin tone.

Table S5: The number of subjects of each Fitzpatrick scale with missing ITA

Fitzpatrick scale	Number of subjects with missed ITA
II	2
III	2
IV	4
V	1
VI	2

- In figure 2, I suggest keeping the same color scheme (ie. Blue for within error range, green for underestimate) as in figure 1 for consistency between the figures.

Thank you for this suggestion. This has been addressed in the updated manuscript Fig 2.

Reviewer #2: Thank you for allowing me to review your study. This manuscript compares the accuracy and reliability of Sp02 measurements from consumer wearable smart devices to a single commercially available pulse oximeter.

Major Comments:
Were these inpatients or outpatients or just patients recruited to a lab? It is not very clear
We would like to thank the reviewer for the opportunity to clarify the characteristics of our study participants. We have updated the *Patient Cohort* sub-section under the *Results* section to state that 30 participants were recruited from outpatient clinics, while 19 were inpatient. In this section we have also added additional patient characteristics as per the request of Reviewer #1 and to improve the comprehensiveness of the cohort description.

The authors should report co-morbid pulmonary disease if available.
As per the previous comment, we have updated the same *Patient Cohort* sub-section of *Results* to include participants' relevant pulmonary and/or cardiovascular disease history, as well their oxygen use.

The authors only use one version of a "gold" standard pulse oximeter to compare to. The "gold standard" should be an arterial blood gas. Also if you were not going to use the "Gold" standard, why just use one model of pulse oximeter, you could have used 2-3 and made an average reading.

We agree that an inherent limitation of this study is the reference device. We chose to use this device because it is the current standard of care used in clinics and therefore is currently the most common method that clinicians use for decision-making. We strongly agree that arterial blood gas would be the optimal gold standard measurement of oxygen saturation, and as such, we refer to the Masimo MightySat as a "reference standard" in our paper [1]. While out of scope of the current work, we now include the use of arterial blood gas as a future direction in the *Discussion* section.

[1] Goldsack et al. Verification, Analytical Validation, and Clinical Validation (V3): The Foundation of Determining Fit-for-Purpose for Biometric Monitoring Technologies (BioMeTs). 3:55 (2020) *NPJ Digital Medicine*.

There are significant interference in the wavelengths by skin pigmentation in different races. A significant limitation to this study is that consumer wearables are compared to a device with known racial bias allows bias into the results which are not accounted for. If the authors had compared the wearable devices to an arterial blood gas, this bias would be taken out of the equation. This should significantly be highlighted in the limitations section.

As in our previous response, we agree that arterial blood gas would be the gold standard measurement tool of oxygen saturation and have added this as a future direction in the *Discussion* section (lines 330-334) and discussed the potential issue of racial bias (line 91-96) and its possible effects on our study's findings (line 334-340).

The Fitzpatrick skin scale is increasing being discovered to not act well in non-White skin types. The limitations of this scale for use in minority patients should be acknowledged.

The Fitzpatrick scale, while an industry standard, is indeed not the best suited tool for the task at hand. We have clarified its limitations on lines 342-348 of the Discussion section. To address the limitations of the Fitzpatrick scale, in this study we also added objectively-measured skin tone using an optical measurement tool, the *Delfin SkinColorCatch,* which reports Individual Typology Angle (ITA) . In the supplement of our paper, we show the high concordance between the Fitzpatrick and ITA measurements. Further, we evaluated whether ITA impacted the outcomes of interest for this study, including the MAE, MDE, Missingness and RMSE between SpO_2 readings from smartwatches and reference standard.
